# The Acute Superficial Siderosis Syndrome — Clinical Entity, Imaging Findings, and Histopathology

**DOI:** 10.1007/s12311-022-01387-3

**Published:** 2022-03-22

**Authors:** Lucie Friedauer, Christian Foerch, Joachim Steinbach, Elke Hattingen, Patrick N. Harter, Moritz Armbrust, Hans Urban, Eike Steidl, Elisabeth Neuhaus, Sophie von Brauchitsch

**Affiliations:** 1 Department of Neurology, University Hospital/Goethe University Frankfurt, Schleusenweg 2-16, 60528 Frankfurt am Main, Germany; 2 Department of Neuro-Oncology, University Hospital/Goethe University Frankfurt, Frankfurt am Main, Germany; 3 University Cancer Center Frankfurt (UCT), University Hospital/Goethe University Frankfurt, Frankfurt am Main, Germany; 4 German Cancer Consortium (DKTK), Partner Site Frankfurt/Mainz, Heidelberg, Germany; 5 German Cancer Research Center (DKFZ), Heidelberg, Germany; 6 Institute of Neuroradiology, University Hospital/Goethe University Frankfurt, Frankfurt am Main, Germany; 7 Neurological Institute (Edinger Institute), University Hospital/Goethe University Frankfurt, Frankfurt am Main, Germany; 8 Epilepsy Center Frankfurt Rhine-Main, Department of Neurology, University Hospital Frankfurt and Goethe University, Frankfurt am Main, Germany

**Keywords:** Siderosis, Ataxia, Cerebellum, Hemosiderin, Acute

## Abstract

Superficial siderosis is a consequence of repetitive bleeding into the subarachnoid space, leading to toxic iron and hemosiderin deposits on the surface of the brain and spine. The clinical and radiological phenotypes of superficial siderosis are known to manifest over long time intervals. In contrast, this study defines the “acute superficial siderosis syndrome” and illustrates typical imaging and histopathological findings of this entity. We describe the case of a 61-year-old male patient who was diagnosed with a melanoma metastasis in the right frontal cortex in February 2019. Within a few weeks he developed a progressive syndrome characterized by cerebellar ataxia, gait disturbance, signs of myelopathy, and radiculopathy. MRI revealed ongoing hemorrhage from the metastasis into the lateral ventricle and the subarachnoid space. A semiquantitative assessment of three subsequent MRI within an 8-week period documented the rapid development of superficial siderosis along the surface of the cerebellum, the brain stem, and the lower parts of the supratentorial regions on T2*-weighted sequences. The diagnosis of a superficial siderosis was histopathologically confirmed by identifying iron and hemosiderin deposits on the cortex along with astrogliosis. The recognition of this “acute superficial siderosis syndrome” triggered surgical removal of the hemorrhagic metastasis. Based on a single case presentation, we define the “acute superficial siderosis syndrome” as a clinical entity and describe the radiological and histopathological characteristics of this entity. Early recognition of this syndrome may allow timely elimination of the bleeding source, in order to prevent further clinical deterioration.

## Introduction

Superficial siderosis is characterized by hemosiderin deposits on the surface of the central nervous system (CNS) resulting from recurrent subarachnoid hemorrhages of different origin. Typically, it is diagnosed by MRI through a hypointense rim on the surface of the CNS in T2*-weighted images [1].

In response to subarachnoid hemorrhages, microglia, and Bergmann-glia in the cerebellar cortex produce hemooxygenase-1 and ferritin. Hemooxygenase-1 converts heme to free iron, which is converted to hemosiderin by ferritin. However, in the context of prolonged or recurrent bleeding, ferritin synthesis is depleted, causing deposits of free iron on the subpial layers of the brain and spinal cord. The accumulation of hemosiderin and free iron around the CNS leads to progressive cell damage [2]. Depending on its distribution with respect to anatomic structures, superficial siderosis can result in different neurological deficits such as gait ataxia, myelopathy, or cranial nerve deficits [3].

Common causes of recurrent subarachnoid hemorrhage include cerebral amyloid angiopathy (CAA), vascular malformations, and spinal dural abnormalities (such as persisting dural leaks) [3–5]. Published reports of superficial siderosis in the context of hemorrhagic brain metastases are limited [6]. In malignant melanoma, brain metastases occur in about 40% of patients with a substantial risk of intratumoral hemorrhage [7, 8].

Depending on the underlying pathology, the clinical and radiological phenotypes of superficial siderosis usually manifest slowly over long time intervals [9]. In contrast, we now present the case of a patient with a cerebral metastasis of a malignant melanoma who developed superficial siderosis in “fast motion” as a consequence of ongoing and extensive intraventricular bleeding followed by a distribution of the blood throughout the CSF spaces. We define this “acute siderosis syndrome” and semiquantitatively measured the progression of the siderosis on brain imaging. In addition, we characterized the histopathological changes on the brain surface resulting from iron and hemosiderin overload. Remarkably, along with clinical deterioration, siderosis progression on brain imaging was the critical finding, triggering surgical removal of the hemorrhagic metastasis.

## Methods

### Clinical Setting

The patient was treated at the Department of Neurology, Goethe-University, Frankfurt am Main, Germany. All clinical data (in particular, the medical history and the neurological examinations) were obtained from the authors of this manuscript in person in the presence of the patient. Written informed consent was received from the patient to present the available data in this single case study.

### Neuroimaging

#### Routine MRI

The patient underwent a number of spinal and cerebral MRI scans, including four consecutive cerebral examinations within 64 days from March to May 2020. The brain MRI examinations were all conducted in a 1.5 Tesla whole-body scanner (Achieva®, Philips Healthcare, Amsterdam, Netherlands) using a dStream HeadSpine ® coil (Philips Healthcare, Amsterdam, Netherlands). The examination protocol included T2- and T1-weighted sequences, a fluid attenuated inversion recovery (FLAIR) sequence, a diffusion weighted sequence (DWI), a time-of-flight angiography (TOF), and contrast enhanced T1-weighted sequences. In addition, these examinations included a susceptibility weighted imaging (SWI) sequence (repetition time 52 ms; echo time 12 ms; flip-angle 20°; field of view 230 × 183 × 130 mm, matrix 260 × 203 × 104, scan time 1:45 min). The spine MR images shown were acquired in May 2020 on a 3 Tesla whole-body scanner (Skyra®, Siemens, Erlangen, Germany) and included sagittal T2- and T1-weighted imaging before and after contrast administration.

#### Quantification of Superficial Siderosis on MRI Scans

MRI data of the four examinations mentioned above was preprocessed using FMRIB’s Software Library 6.0 [10]. To analyze the dynamics of siderosis development, the SWI images were linearly co-registered to the 3D T1-weighted image of the first timepoint (March 2020) [11]. The 3D T1-weighted image was skull-stripped, and the brain extraction mask was applied to the SWI images after manual correction (which was necessary mainly because of cerebral metastasis). The patient’s total brain volume was defined as the volume of the brain extraction mask. Intensity histograms with 200 bins, covering the range of the data (intensity values of 0 to 2050, arbitrary units) were created for all four masked SWI images using python (version 3.7) and matplotlib (version 3.3). To identify hypointense voxels that appear black in a standard windowing (representing siderosis as well as calcifications and blood vessels), an intensity range was defined, starting from 0 and ending at 1000. The number of “black-appearing” voxels was then calculated for all four histograms. Finally, the percentage increase of “black-appearing” voxels was assessed using the first examination as a baseline as well as the affected brain volume in relation to the total brain volume. As vessels and calcifications should be stable over the observed short time period, changes in the number of black voxels can mostly be attributed to the increasing hemosiderin deposits.

### CSF Analysis

The patient underwent three lumbar punctures under standardized conditions. Standard CSF analyses included cell count, total protein, lactate, glucose, and cytological analysis. Leukocyte count ≤ 5/µl, red blood cells count = 0/µl, total protein < 450 mg/l, lactate 1,1–2,4 mmol/l, and glucose 40–70 mg/dl were considered as normal findings.

### Histopathological Analysis

The patient underwent surgical removal of the hemorrhagic melanoma metastasis. The tissue was fixed in 4% paraformaldehyde for 24 h, paraffin-embedded and subsequently cut to a thickness of 4 µm, followed by clearing in xylene and rehydration with decreasing concentrations of ethanol. Sections were stained with hematoxylin and eosin by established procedures as well as with Perl’s Prussian Blue stain for iron. For the Perl’s stain, the sections were incubated in 10% potassium ferrocyanide(II) solution for 5 min. Immediately before use, equal parts of 20% hydrochloric acid and 10% potassium ferrocyanide(II) were mixed, followed by a 30-min immersion of the sections in this solution. The sections were washed in distilled water, counterstained with nuclear fast red for 5 min, washed in distilled water again and subsequently dehydrated in an ascending alcohol series followed by processing with xylene and coverslipping.

For immunohistochemistry analyses, the Bond™ III Fully Automated IHC/ISH stainer (Leica Biosystems) was used. Within the stainer, the sections were de-waxed using the Bond™ Dewax Solution (Catalog #AR9222) and subsequently rehydrated in a decreasing ethanol series. A heat-induced antigen retrieval was applied to the slides which were exposed to an EDTA-based epitope retrieval solution (Epitope Retrieval Solution 2, Catalog #AR9640) and heated to 100 °C for 10 or 20 min subject to the specific primary antibody used. Applying the Bond™ Polymer Refine Detection (Catalog # DS9800), the sections were applied to a peroxide block using hydrogen peroxide to quench endogenous peroxidase activity, followed by the application of one of the following antibodies: glial fibrillary acidic protein (GFAP, polyclonal rabbit, 1:14,000 dilution, DAKOCytomation, Denmark), and CD68 (monoclonal mouse, 1:200 dilution, DAKOCytomation, Denmark). Depending on the antigen of interest, the sections were exposed to either post-primary rabbit anti-mouse IgG linker reagent for localization of mouse antibodies followed by incubation with the anti-rabbit Poly-HRP-IgG reagent for localization of rabbit antibodies or incubated with the anti-rabbit Poly-HRP-IgG reagent only. Subsequently, the substrate chromogen, 3,3′-diaminobenzidine tetrahydrochloride hydrate (DAB) was applied to the sections for complex-visualization. Lastly, a hematoxylin counterstaining was conducted for the visualization of cell nuclei with subsequent blueing and application of the slides to an ascending ethanol series and xylene, followed by coverslipping. The slides were imaged with a bright-field microscope.

## Results

### Case Report and Clinical Findings

**Fig. 1:**
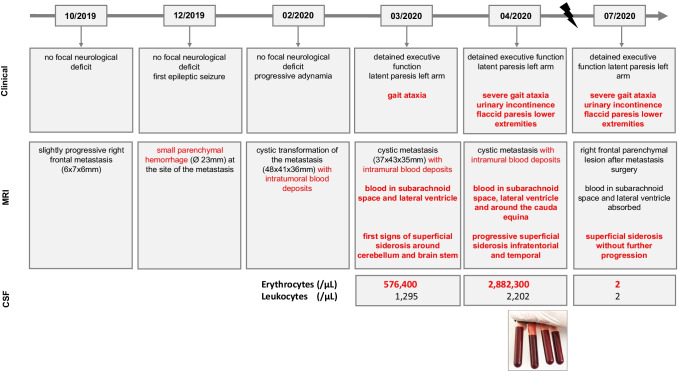
Timeline including clinical findings, MRI findings and results of the CSF analysis. The lightning sign in the timeline indicates the time point of surgical removal of the hemorrhagic melanoma metastasis (end of May 2020). Blood accumulation within the metastasis on MRI is described in thin red color. Blood entering the subarachnoid space and the ventricular system including the subsequent development of superficial siderosis and the corresponding clinical symptoms are described in bold red color. Based on this color coding, the dynamics of the bleeding event (separately from the development of the metastasis itself) in terms of clinical findings, development of superficial siderosis and CSF results can be extracted from the figure

The 61-year-old Caucasian male patient was diagnosed with a malignant melanoma with pulmonary metastasis at the age of 58 years. During a pause from checkpoint inhibitor therapy (ipilimumab and nivolumab) due to medication-induced autoimmune pneumonitis, the patient developed four cerebral metastases at the age of 59, which were treated by a single session of radiosurgery (05/2019). While the reinstalled maintenance therapy with nivolumab resulted in a complete remission and three of the cerebral metastases resolved after radiosurgery, the right frontal lobe metastasis underwent a post-therapeutic pseudoprogression (10/2019; Fig. 1).

In 12/2019, the patient was hospitalized due to a first generalized tonic–clonic seizure. The neurological exam continued to be unremarkable. Cerebral MRI showed a primary event of cerebral hemorrhage localizing around the right frontal metastasis. The follow-up MRI in 02/2020 depicted the cystic transformation of the metastasis with an intratumoral blood deposit. While having developed a progressive adynamia at that time, the patients still did not present with any neurological deficit.

**Fig. 2:**
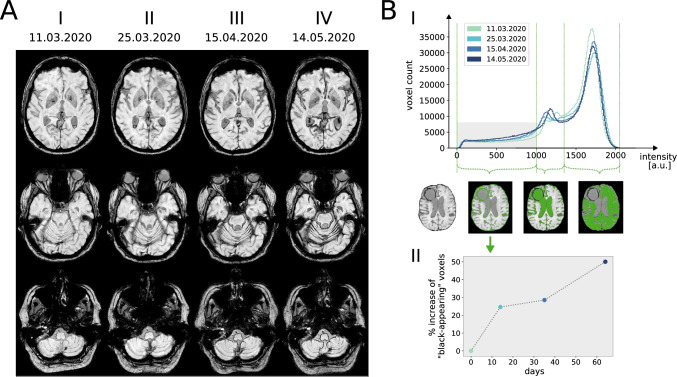
**A** Columns I–IV show corresponding slices (susceptibility weighted imaging, SWI) of four brain MRI scans that were acquired over a 64-day period from March to May 2020. The progression of the superficial sideroses is visible as a black coating of the brain surface most pronounced on the top of the cerebellum and around the brainstem. **B** Panel I shows the intensity histograms derived from the susceptibility weighted imaging (SWI) sequences of the four brain MRI scans. The histogram shows two main peaks that mostly correspond to brain tissue (right peak) and cerebrospinal fluid (left peak). The gray box in the lower left corner of the plot depicts the intensity range for”black-appearing” voxels. Voxels within the different intensity ranges are masked in green in the SWI image below the x-axis for illustration. As a reference, the corresponding SWI image without mask is shown on the left. Derived from the intensity range within the gray box panel II shows the percentage increase of “black-appearing” voxels relative to the first MRI scan over time

One month later (3/2020), the patient presented to our center with detained executive functions and a latent paresis of the left upper extremity with minor spasticity which were considered a consequence of metastasis progression in the right frontal cortex. Remarkably, a pronounced gait ataxia was apparent limiting his walking distance to approximately 50 m. At this time, MR imaging identified both acute and subacute hemorrhages in the subarachnoid space and the right lateral ventricle, indicating repetitive or continuous metastatic bleeding. First signs of superficial siderosis were apparent around the cerebellum and the brain stem (Fig. 2A, I). Lumbar punctures confirmed the presence of large amounts of blood in the cerebrospinal fluid. Since asymptomatic DWI lesions were detected in cranial MRI, the presence of an additional (checkpoint inhibitor therapy-associated) vasculitis was discussed, which may have contributed to increased metastatic bleeding. The assumed vasculitis was treated with high-dose cortisone therapy and infliximab. A subsequent pathological examination could not confirm the presence of vasculitis with certainty. Following in-hospital rehabilitation measures, the patient was discharged home with optimized domestic care.

Six weeks later, admission (4/2020) resulted from the development of a progressive gait disturbance binding the patient to a wheelchair for 3 weeks, and from urinary incontinence for about a week. On neurological examination, the patient presented with a mid-grade paresis of his lower extremities (hip flexion 4/5, knee flexion 3/5, knee extension 3/5) with loss of deep tendon reflexes and a severe urinary incontinence. In addition to the hemorrhage into the right frontal metastasis, progressive superficial siderosis coating large parts of the brain (with an infratentorial and temporal focus) and the spinal cord was detected (Figs. 2A, II–IV, and 3). In addition, blood sedimentation levels in the ventricles and surrounding the cauda equina were identified. As before, strongly stained hemorrhagic cerebrospinal fluid was drawn in the following lumbar puncture.

**Fig. 3:**
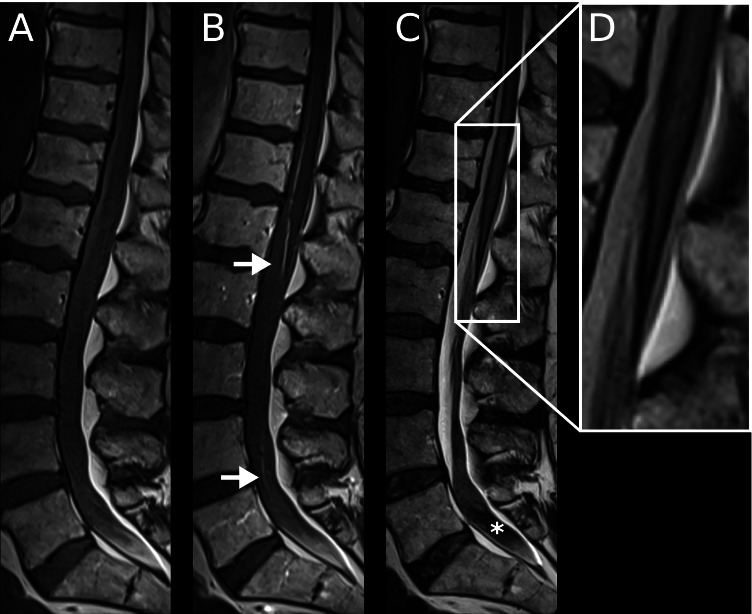
Sagittal pre- **A** and post-contrast **B** T1-weighted MR images of the spinal cord, acquired May 2020, show abnormal linear enhancement along the cord surface and diffuse enhancement of the nerve roots of the cauda equina (B, →). Sagittal T2-weighted images **C** and **D** show intramedullary cord hyperintense edema and hypointense hemosiderin deposition along the spinal cord surface. The thecal sac shows hypointense blood-fluid levels **C**,*

It was assumed that the toxicity of the iron deposits on the surface of the brain and myelon caused by the ongoing massive blood overload in the subarachnoid space provokes progressive neurological dysfunction. Therefore, the patient was advised to undergo complete resection of the right frontal cerebral metastasis to remove the source of bleeding.

After surgery (end of May 2020), the patient stayed on intensive care unit for 3 weeks, before being stepped down to normal ward. On re-examination, 2 months after surgery, the patient presented with a stable mid-grade flaccid paresis of his lower extremities as well as persisting urinary incontinence. Gait was still highly unstable. No further deterioration of the clinical syndrome occurred. Cerebral and spinal MRI showed stable findings of superficial siderosis. However, CSF diagnostics revealed a normalized erythrocyte count. Increased lactate levels and a blood-CSF-barrier dysfunction were still present (Fig. 1).

### Neuroradiological Findings

Over a period of about 2 months, repetitive cerebral MRI examinations revealed a massive progression of superficial siderosis. Figure 2A depicts how the siderosis became visible on SWI as hypointense (“black-appearing”) coating of increasing thickness within the ventricles and on the surface of the brain. While the distribution is mostly symmetric, an accentuation on the top of the cerebellum and around the brainstem was apparent. In order to visualize and quantify the dynamics of siderosis progression, we calculated the volume increase of the siderosis in relation to the patient’s total brain volume (1537 cm^3^). Setting the first examination as a baseline, Fig. 2B illustrates that 8.0% of the total brain volume was involved within the observed period of 64 days. While this translates to an average volume of 1.9 cm^3^ becoming affected on SWI imaging per day, the graph in Fig. 2B rather suggests a relapsing than a continuous pattern of the underlying bleeding. Additional MRI of the spine further substantiated the extent of the bleeding, showing not only superficial siderosis of the spinal cord but also a marked clouding of the CSF and sedimentation of blood products in the lumbar spinal canal (Fig. 3).

### Laboratory Findings

First CSF analysis was performed in March 2020. Erythrocytes were massively increased (576,400/µl). Cytological evaluation revealed hemophagocytosis at different stages (erythrophages, erythrohemosiderophages and hemosiderophages as well as cells of the peripheral blood). In addition, increased lactate (5.13 mmol/l) and a blood-CSF-barrier dysfunction (total protein 3.06 mg/l) were observed. CSF-analysis performed in April 2020 showed a further increase in lactate and the number of erythrocytes. Malignant cells were not detected in both lumbar punctures. In July 2020, CSF findings have been fully normalized (Fig. 1).

### Neuropathological Findings

**Fig. 4:**
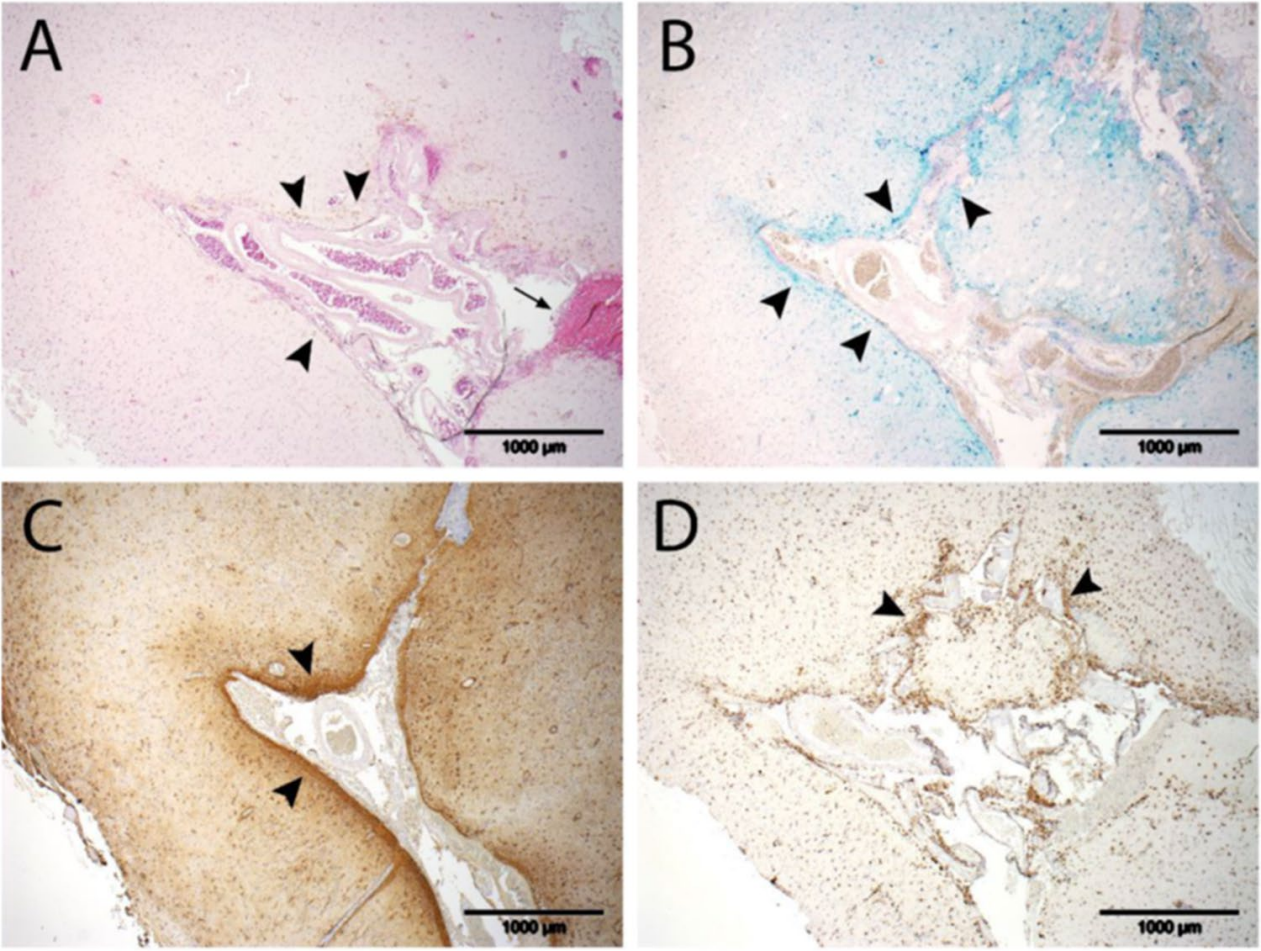
Representative images of **A** hematoxylin and eosin staining, **B** Perls Prussian blue staining, **C** GFAP and **D** CD68 immunohistochemistry of superficial cortical tissue from the right frontal lobe. **A** Apart from recent bleeding constituents within the leptomeningeal compartment (arrow), hemosiderin deposits can be seen within the molecular layer of the cortex (arrowheads). **B** Covering the superficial layers of the cortex, ribbon-like iron deposits are appreciable (arrowheads). **C** Within the superficial cortex area, iron deposits are accompanied by reactive astrocytes (arrowheads). **D** Superficially located within the cortex, CD68-positive macrophages can be observed (arrowheads)

Following surgical removal of the hemorrhagic melanoma metastasis, histopathological analysis depicted iron deposits covering the superficial layer of the cortex as well as hemosiderin deposits in the molecular layer of the cortex. Combined with the reactive astrocytes and the CD68-positive macrophages in the superficial layer of the cortex as signs of astrogliosis and immune response, these findings are in accordance with iron toxicity and the diagnosis of a recently developed superficial siderosis (Fig. 4).

### Definition of the Acute Superficial Siderosis Syndrome

**Fig. 5:**
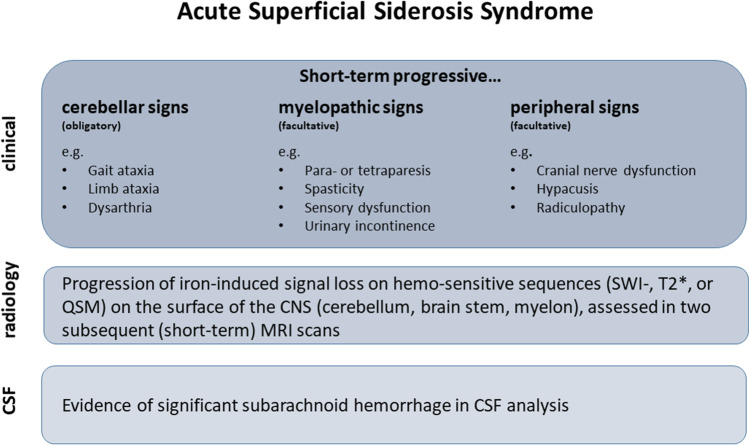
Definition of the acute superficial siderosis syndrome based on clinical, imaging, and laboratory findings

Based on the characteristic clinical syndrome of patients with infratentorial superficial siderosis and its short-term progression in case of ongoing subarachnoid hemorrhage, we suggest the definition of an “acute superficial siderosis syndrome” (Fig. 5). Given that the cerebellum is particularly susceptible for siderosis development, cerebellar signs may be considered obligatory in patients with infratentorial siderosis, whereas signs of myelopathy and symptoms associated with cranial nerve dysfunction or radiculopathy may be considered facultative. Evidence of siderosis progression on short-term follow-up MR images (SWI-sequence) should be an essential requirement for diagnosing this syndrome.

## Discussion

The reported case is remarkable for several reasons: First, this acute form of superficial siderosis with rapidly progressive neurological deterioration has not been described before. Therefore, this case sets an example for tracing the typical clinical, neuropathological, and neuroradiological characteristics of superficial siderosis in “fast motion”. Until now, superficial siderosis was understood to develop slowly over an extended time period from months to years [3]. Second, we provide a description of tissue reaction on a neuropathological level and developed a method for rapid semiquantitive assessment of siderosis progression (the “coating”) on brain MRI. Third, we depict that the early recognition of acute siderosis development is of importance to prevent progressive neurological deficits resulting from iron-associated toxicity by removing the bleeding source.

Previous studies have shown that superficial siderosis is usually associated with a slowly progressive clinical syndrome. Few case reports describe fast onset of neurological deficit in the context of superficial siderosis, e.g., in patients with intracranial hypotension due to traumatic CSF leaks and subsequent cerebellar hemorrhage and siderosis [12]. Superficial siderosis results from repetitive subarachnoid hemorrhage over an extended period of time (as in CAA or spinal dural leaks) [5, 9]. Hemolytic enzymes (including hemooxygenases) are particularly induced in Bergmann-glia and microglia of the cerebellar cortex, making infratentorial brain structures susceptible for siderosis development [2]. In consequence, infratentorial superficial siderosis preferably develops on the cerebellar cortex and is associated with a very characteristic clinical syndrome including gait ataxia, cranial nerve deficits, and myelopathy [1, 3]. Besides the development of gait ataxia, our case presented with a pronounced affection of the spinal cord indicated by a rapidly advancing paraparesis and urinary incontinence. Notably, signs of a flaccid paresis were apparent, pointing to additional radiculopathy resulting from large amounts of blood sedimented around the cauda equina.

The exact pathophysiological mechanisms behind the fast-motion siderosis in opposition to a slowly progressive one have not been characterized so far. The continuous bleeding over a relatively short period of time in case of metastatic hemorrhage stands in contrast to the sporadic bleeding in patients with CAA and spinal dural pathologies. As a consequence of the large amount of blood that entered the CSF in our case, rapid depletion of enzymatic hemolytic processes (including the conversion of heme to iron and the conversion of iron to hemosiderin) can be assumed. This results in a “chaotic” deposition of free iron on the subpial layers of the brain and spine with subsequent astrogliosis and a corresponding histiocytic response, as detected in the histopathological analysis. Free iron is known to be neurotoxic, both for the CNS and for the peripheral nervous system [13, 14].

While previous studies demonstrated a slow progression of superficial siderosis pattern on subsequent cerebral MR images (consistent with a slowly progressive clinical syndrome) [15], repetitive MRI scans in our case revealed a massive signal loss in T2*-weighted sequence due to increasing iron deposition in the surface of the cerebellum, the brain stem, and the spinal cord within a short period of time (approximately 2 months). Remarkably, all 4 consecutive scans were acquired using the same MR scanner with identical image processing techniques. This allowed us to develop a semiquantitative method to determine the dynamics of siderosis accumulation over time. Together with the characteristic clinical syndrome and the evidence of significant CSF bleeding in lumbar puncture, the iron induced signal loss on MR imaging in short intervals is considered an essential feature for diagnosing an “acute siderosis syndrome.” In the future, quantitative susceptibility mapping may help to precisely monitor the dynamics of superficial siderosis development, both over shorter and longer periods of time [16].

In our case, the rapid development of the clinical syndrome combined with the rapid progression of superficial siderosis in cerebral MRI finally triggered surgical removal of the right frontal metastasis by craniotomy. Clinical and imaging findings related to the superficial siderosis stabilized after stopping the bleeding source. However, hemosiderin and iron that have already accumulated on the cortex will hardly be removed by endogenous clearing processes. Thus, ongoing neurotoxicity will likely prevent the patient from significant improvement of clinical symptoms. Whether treatment with iron chelators is effective in this context needs to be determined in future studies [15]. In consequence, it is important to recognize the clinical and imaging pattern of the acute superficial siderosis syndrome as early as possible, in order to treat the underlying bleeding source and to avoid subsequent disability accumulation.

As a limitation, this patient did not suffer from acute superficial siderosis development only but had a malignant melanoma with a cerebral metastasis, complicating the assignment of clinical symptoms to the different pathologies. However, ongoing subarachnoid hemorrhage is not likely to develop without an underlying intracranial disease, making this constellation not unusual. Furthermore, in our case, clinical signs of cerebellar, spinal and radicular dysfunction together with the CSF- and imaging findings are not likely to originate directly from the cerebral metastasis in the right frontal cortex. In addition, CSF findings have fully normalized after extirpation of the metastasis, which appears typical for post-hemorrhagic stages but untypical for any other (untreated) disease in the spinal canal (such as carcinomatous meningitis).

In conclusion, superficial siderosis is no longer characterized by an exclusively slowly progressive clinical phenotype. Superficial siderosis in “fast motion” can be diagnosed based on a characteristic neurological syndrome supported by siderosis progression on semiquantitative MRI analysis. An early recognition of the acute superficial siderosis syndrome is of relevance in order to stop the bleeding source and to prevent further clinical deterioration.
